# Model-Optimizing Radiofrequency Parameters of 3D Finite Element Analysis for Ablation of Benign Thyroid Nodules

**DOI:** 10.3390/bioengineering10101210

**Published:** 2023-10-17

**Authors:** Fabiano Bini, Andrada Pica, Franco Marinozzi, Alessandro Giusti, Andrea Leoncini, Pierpaolo Trimboli

**Affiliations:** 1 Department of Mechanical and Aerospace Engineering, Sapienza University of Rome, 00184 Rome, Italy; andrada.pica@uniroma1.it (A.P.); franco.marinozzi@uniroma1.it (F.M.); 2 Department of Biomedical Sciences, University of Sassari, 07100 Sassari, Italy; 3 Dalle Mole Institute for Artificial Intelligence (IDSIA), Università della Svizzera Italiana (USI), The University of Applied Sciences and Arts of Southern Switzerland (SUPSI), 6900 Lugano, Switzerland; alessandro.giusti@idsia.ch; 4 Servizio di Radiologia e Radiologia Interventistica, Istituto di Imaging della Svizzera Italiana (IIMSI), Ente Ospedaliero Cantonale (EOC), 6900 Lugano, Switzerland; andrea.leoncini@eoc.ch; 5 Clinic of Endocrinology and Diabetology, Lugano Regional Hospital, Ente Ospedaliero Cantonale (EOC), 6500 Bellinzona, Switzerland; 6 Faculty of Biomedical Sciences, Università della Svizzera Italiana, 6900 Lugano, Switzerland

**Keywords:** thyroid benign nodules, radiofrequency ablation, finite element method, optimization model, treatment planning

## Abstract

Radiofrequency (RF) ablation represents an efficient strategy to reduce the volume of thyroid nodules. In this study, a finite element model was developed with the aim of optimizing RF parameters, e.g., input power and treatment duration, in order to achieve the target volume reduction rate (VRR) for a thyroid nodule. RF ablation is modelled as a coupled electro-thermal problem wherein the electric field is applied to induce tissue heating. The electric problem is solved with the Laplace equation, the temperature distribution is estimated with the Pennes bioheat equation, and the thermal damage is evaluated using the Arrhenius equation. The optimization model is applied to RF electrode with different active tip lengths in the interval from 5 mm to 40 mm at the 5 mm step. For each case, we also explored the influence of tumour blood perfusion rate on RF ablation outcomes. The model highlights that longer active tips are more efficient as they require lesser power and shorter treatment time to reach the target VRR. Moreover, this condition is characterized by a reduced transversal ablation zone. In addition, a higher blood perfusion increases the heat dispersion, requiring a different combination of RF power and time treatment to achieve the target VRR. The model may contribute to an improvement in patient-specific RF ablation treatment.

## 1. Introduction

The thyroid gland plays a vital role in the endocrine system. Disorders affecting its function and structure require accurate treatment planning for optimal patient outcomes. Thyroid nodules represent an increase in thyroid volume with overgrowth and structural or functional transformations of one or more areas of the thyroid gland [1]. Benign thyroid nodules are a prevalent clinical problem that affects a significant portion of the population [2,3]. According to studies conducted on adults living in iodine-sufficient areas, approximately 4–7 percent of women and 1 percent of men have palpable thyroid nodules [4]. Nonetheless, when estimated with ultrasound examination, the prevalence of nodules is reported in up to 40–50 percent of the population [5]. Symptomatic cases among the patients with thyroid nodules are low [4].

Until now, total or partial thyroid surgery represents the predominant therapeutic method for symptomatic thyroid nodules [5]. However, in recent years, non-surgical options to treat benign thyroid nodules, e.g., laser [6], radiofrequency [7,8,9,10], microwaves [11], and high intensity focused ultrasound [12,13,14,15], have emerged as promising treatment alternatives to surgery. Radiofrequency (RF) ablation is an ultrasound-guided, minimally invasive procedure that uses thermal energy to ablate thyroid nodules. RF ablation offers several advantages over surgical interventions, such as reduced invasiveness, shorter recovery time, and preserved thyroid function [16]. To date, several academic societies in various countries, e.g., Korea, Italy, United Kingdom, and Austria, have formulated clinical practice guidelines, opinion statements, or recommendations regarding the use of RF ablation as a thyroid nodule treatment [17].

During RF ablation, a thin needle electrode is inserted into the thyroid nodule under ultrasound guidance. Radiofrequency energy is then delivered through the electrode, generating heat and causing coagulative necrosis within the nodule [18]. The heat destroys the nodular tissue while sparing the surrounding healthy thyroid tissue. The ablated tissue is gradually absorbed and replaced by fibrosis over time, leading to nodule shrinkage [19].

Numerous studies have demonstrated the efficacy of RF ablation in reducing the volume of benign thyroid nodules [1,5,7,18,19]. The reported nodule volume reduction ranges from 50 percent to 80 percent following RF ablation treatment [19]. Furthermore, RF ablation can reduce or eliminate the need for thyroid hormone replacement therapy with respect to partial thyroid surgery (i.e., hemithyroidectomy).

Factors, such as nodule size, initial volume, and treatment energy delivered, play a role in determining the achieved volume reduction rate (VRR). For instance, single RF ablation session is efficient for small- and medium-sized thyroid nodules, achieving a volume reduction ratio of up to 80 percent according to [20]. Conversely, larger nodules tend to have lower VRRs compared to smaller ones. Lin and colleagues [5] reported in their retrospective study that large solid thyroid nodules may require multiple sessions of RF ablation to achieve nearly complete ablation. Moreover, Deandrea and colleagues [7] performed a retrospective study involving 115 patients with benign thyroid nodules treated by means of RF ablation. They reported a significant reduction in nodule volume, in the range 50–75 percent, and 87 (75.7 percent) nodules showed a VRR above 50 percent [7]. 

Several investigations were performed to identify the significant parameters that may influence the RF ablation treatment. In this context, Trimboli et al. [8] showed that the energy delivered with RF ablation is the only technical parameter significantly correlated with the VRR of thyroid nodules. Deandrea et al. [7] analysed the correlations of different parameters, e.g., power and energy, with VRR. The authors of [7] concluded that taking into account the baseline nodular volume and delivering the adequate energy per volume may allow the optimization of the treatment.

In this study, we develop a computational model in order to simulate and optimize RF ablation procedures on thyroid benign nodules, in order to contribute to treatment planning. In recent decades, computational models have highly contributed to the improvement of the medicine field by providing valuable tools to simulate and analyse complex physiological processes [21,22], aiding in treatment planning [23,24], dose optimization [25], and treatment response prediction [10]. There exists a substantial body of literature that simulates RF ablation treatment by means of finite element (FE) models. On this topic, a comprehensive review was conducted by Singh and colleagues [23]. A majority of prior FE studies concerning RF ablation were focused on analysing the liver tissue. These studies [22,23,26,27] investigated mostly the dependence of injury profiles of the liver tissue-specific factors, e.g., tissue perfusion, material properties, and disease state, that were found to affect ablative therapy. It is evident from [22] that it is important to develop organ-specific numerical simulations of RF treatment to provide a priori information about possible outcomes and risks before the onset of clinical RF therapy.

Compliant with the above facts and to the best of our knowledge, in the available literature, only Jin and co-workers [28] developed a 3D FE model to predict the effects induced by RF thermal ablation within thyroid tissues. However, contrary to our study, their model focused on two treatment strategies related to the electrode system, i.e., single-cooled-electrode modality and two-cooled-electrode system. In addition, other studies considered the development of an FE model of the thyroid, but the simulations were focused on a different treatment method, e.g., focused ultrasound [29,30]. Other publications focused on the development of FE models of the neck region for the analysis of bioheat transfer in the human neck and subsequent comparison with images achieved with infrared thermography [25,31,32].

In silico studies also enable the exploration of various treatment scenarios, optimizing therapeutic outcomes while minimizing risks [33]. In this work, the FE model aims to optimize input parameters of RF ablation treatment in order to achieve a specific VRR for a benign thyroid nodule by minimizing treatment time and avoiding damage to surrounding healthy tissues. The FE analysis allows to predict the effect of RF ablation treatment within the 3D volume of the tissue. The working conditions considered here, e.g., the size of the benign thyroid nodule and the frequency of the thermal therapy, are set in order to mimic the clinical study by Deandrea and colleagues [7]. The efficiency of RF electrodes with different active tip lengths and the influence of tumour blood perfusion rate on RF ablation were also explored. Computational models offer a platform for modelling in silico studies and allow for personalized treatment strategies based on patient-specific characteristics [24,25]. This study also aims at setting the basis for the development of a predictive model for RF treatment parameters that can be personalized for each patient.

## 2. Materials and Methods

### 2.1. Geometric Model

**Figure 1:**
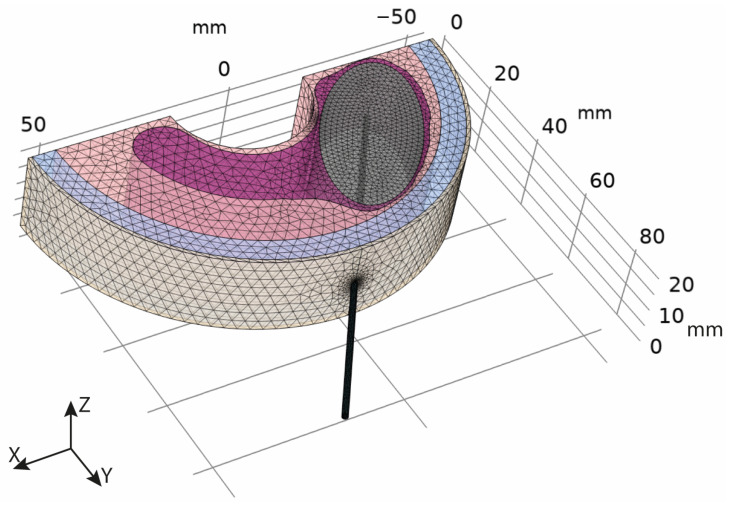
A 3D meshed model of the neck region comprising skin (light yellow), fat (light blue), muscle (light pink), thyroid (magenta), and a nodule (grey) within the thyroid.

A three-dimensional (3D) FE model of the neck region is implemented. The simulated neck comprises skin, fat, muscle, thyroid, and an elliptic nodule within the thyroid lobe (Figure 1). The thickness of the different anatomical regions is in agreement with previous studies [31,34,35]. Specifically, we set a thickness of 1 mm for the skin, 6 mm for the fat region, 30 mm for the muscle, and 20 mm for the thyroid. The dimensions of the thyroid nodule are within the ranges reported in the available literature [28,31] and are set in order to achieve a volume of the nodule equal to 20 mL, as in the study by Deandrea et al. [7].

The simplified geometry is modelled by an extremely fine tetrahedral mesh with roughly 100 k elements. Thus, the model is characterized by approximately 1 × 10^6^ degrees of freedom. 

The RF ablation treatment is simulated by considering a RF electrode inserted into the thyroid tissue up to the nodule. The electrode is placed parallel to the XY plane of the coordinate system, as reported in Figure 1, and it is assumed to be in close contact with the surrounding tissues. Following the experimental study by Deandrea and colleagues [7], we consider a 18-gauge RF electrode with a shaft length of 100 mm. The influence of the electrode characteristics on the RF ablation treatment outcome is analysed by implementing a parametric study of the active tip length of the electrode in the interval from 5 mm to 40 mm at the 5 mm step.

### 2.2. Mathematical Model

RF ablation is modelled as a coupled electro-thermal problem wherein the electric field is applied to induce tissue heating. The electric problem is solved by the Laplace equation [22]:

(1) $$\nabla \cdot \left(\sigma \nabla V\right)=0$$

where σ is the electric conductivity (S/m), V is the electric potential (V), and ∇ is the gradient operator. Quasi-static approximation is adopted since the wavelength of RF current in tissues is greater than the length of active tip and depth of human body [22].

The simulation assumes RF delivery at 480 kHz frequency as in [7,8]. Power and treatment duration are optimized during the simulation in order to achieve a target value of the VRR.

The coupled thermal-electric model, derived from Pennes bioheat equation [22], is utilized to calculate the temperature distribution within the neck region, following Equation (2):

(2) $$\rho C_{p}\partial T/\partial t+\nabla \cdot \left(-k\nabla T\right)=Q_{p}+{\rho_{b}\cdot \omega }_{b}\cdot C_{b}\cdot \left(T_{b}-T\right)+Q_{met}$$

where ρ is tissue density (kg/m^3^), C_p_ is the specific heat of tissue (J/kg∙°C), T is the tissue temperature (°C), k is the thermal conductivity of the tissue (W/m °C), T_b_ is the blood temperature and is set to 37 °C, and ${\rho_{b},\omega }_{b},C_{b}$ are density, perfusion rate (s^−1^), and specific heat of blood, respectively. Q_met_ is the metabolic heat generation.

The volumetric heat generation rate Q_p_ (W/m^3^) due to electrical heating is provided by [22]:

(3) $$Q_{p}=J\cdot E=\sigma {\left|\nabla V\right|}^{2}$$

where J is the current density (A/m^2^), and E is the electric field (V/m).

The tissue damage of the simulated ablation zone is calculated considering the Arrhenius equation (Equation (4)) [21]:

(4) $$D\left(t\right)=A\int_{0}^{t}e^{\frac{-∆E}{RT(\tau )}}d\tau$$

where D(t) is an indicator of the damaged tissue, A is the frequency factor (s^−1^), ∆E is the activation energy for the irreversible damage reaction (J/mol), R is the universal gas constant (J/mol∙K), and T is the absolute temperature (K).

From Equation (4), an estimation of the fraction of tissue damage (θ) can be achieved as follows [24]:

(5) $$\theta =1-e^{-D(t)}$$

where *θ* is a dimensionless factor that ranges between 0 and 1, with *θ* = 1 corresponding to the complete damage.

Dirichlet boundary conditions concerning temperature field of all external boundaries are set as follows:

(6) T=37 °C

Thermal convection with the environment at skin surface is also considered based on study [31]:

(7) $$-n\cdot q=h(T_{ext}-T)$$

where q is the heat flux, h is the convective coefficient equal to 10 (W/m^2^∙K), and the external temperature (T_ext_) is set to 25 °C.

### 2.3. Model Parameters

Table 1 lists the physical parameters considered in the model for skin, fat, muscle tissue, thyroid tissue, benign nodule, and blood, respectively.

It has been demonstrated that the magnitude of the blood perfusion ω varies with the tissue status during the RF ablation process. The blood perfusion rate is assumed to vary as a function of tissue damage as in [28]:

(8) $$\omega_{b}\left(T\right)=\omega_{b0}/e^{D(t)}$$

It is assumed that the increase in electrical conductivity (σ) of p_i_ = 1.5 percent per degree centigrade for healthy and tumoral tissues [22,23,36]:

(9) $$\sigma =\sigma_{ref}\left(1+p_{i}(T-T_{ref})\right)$$

where σ_ref_ is the reference electrical conductivity of the tissue, p_i_ = 1.5 percent and is the percentage of increase, in agreement with [36], T is the temperature, and T_ref_ = 37 °C is the body temperature.

**Table 1:** Physical parameters of each tissue and thyroid nodule employed in the computational model.

		Skin	Fat	Muscle	Thyroid	Nodule
**Thermal conductivity**	k (W/m∙K)	0.37 [31,37]	0.21 [31,37]	0.49 [31,37]	0.52 [31,37]	0.89 [31]
**Density**	ρ (kg/m^3^)	1109 [31,37]	911 [31,37]	1090 [31,37]	1050 [31,37]	1050 [31]
**Specific heat at constant pressure**	c_p_ (J/kg∙K)	3391 [31,37]	2348 [31,37]	3421 [31,37]	3609 [31,37]	3770 [31]
**Electrical conductivity**	σ (S/m)	4.09∙10^−3^ [37]	4.37∙10^−2^ [37]	4.43∙10^−1^ [37]	5.64∙10^−1^ [37]	4.81∙10^−1^ [38]
**Relative permittivity**	ε_r_ (-)	1.06∙10^3^ [37]	57.3 [37]	3.77∙10^3^ [37]	2.18∙10^3^ [37]	2.18∙10^3^ [37]
**Blood perfusion**	ω_b_ (s^−1^)	0.00196 [39]	5.01∙10^−4^ [39]	7.08∙10^−4^ [39]	0.098 [39]	0.0096 [31] 0.021 [40]
**Metabolic heat**	Q_m_ (W/m^3^)	1829.85 [31]	464.61 [31]	1046 [31]	4200 [31]	42,000 [31]
**Frequency factor**	A (s^−1^)	4.575∙10^72^ [41]	4.43∙10^16^ [24]	2.94∙10^39^ [42]	7.39∙10^39^ [28]	7.39∙10^39^ [28]
**Activation Energy**	ΔE (J/mol)	4.71∙10^5^ [41]	1.3∙10^5^ [24]	2.596∙10^5^ [42]	2.577∙10^5^ [28]	2.577∙10^5^ [28]

Thermal conductivity varies as follows:

(10) $$k=k_{ref}+r_{i}\left(T-T_{ref}\right)$$

where k_ref_ is the reference thermal conductivity of the tissue, r_i_ = 0.0013 is a parameter that approximates the temperature dependence of k as in [36], T is the temperature, calculated during the simulation, and T_ref_ = 37 °C is the body temperature. 

Table 2 lists the physical parameters considered for the RF electrode in agreement with the available literature [23,31].

**Table 2:** Physical parameters of the RF electrode.

		Electrode Active Tip	Electrode Shaft
**Thermal conductivity**	k (W/m∙K)	400	0.026
**Density**	ρ (kg/m^3^)	8960	1150
**Specific heat at constant pressure**	c_p_ (J/kg∙K)	385	1700
**Electrical conductivity**	σ (S/m)	5.99∙10^7^	1∙10^−5^
**Relative permittivity**	ε_r_ (-)	1	1

### 2.4. Optimization Model

An optimization algorithm is introduced in order to determine the appropriate power value and duration of the RF ablation treatment. To this end, the BOBYQA (Bound Optimization BY Quadratic Approximation) algorithm is adopted [43]. BOBYQA is a derivative-free iterative algorithm used for finding the minimum value of a function f, subject to constraints on the variables. Specifically, in this model, the objective function is expressed as follows:

(11) $$f={\left(VRR-VRR_{target}\right)}^{2}$$

where VRR_target_ is the target value of the volume reduction rate to be obtained at the end of the treatment. In the present study, the VRR_target_ is equal to 65 percent following the outcomes highlighted in the study by Deandrea and colleagues [7].

The volume reduction rate (VRR) achieved after the RF ablation treatment is calculated as in Equation (12):

(12) $$VRR=\frac{initial\;nodule\;volume-final\;nodule\;volume}{intial\;nodule\;volume}\cdot 100$$

The input parameters that are adjusted during the optimization process include the power and the treatment duration. It is assumed that the RF ablation power can achieve values in the interval between 45 W and 60 W, as mentioned in [7], while the RF ablation treatment can last up to 20 min [7]. In addition, a constraint is also implemented for the temperature reached around the RF needle. In order to avoid the damage of healthy tissues, at distances higher than 10 mm from the RF needle, the temperature should be inferior to 100 °C [10,44]. The algorithm continues the optimization process with adjusted input parameters until the objective function is minimized.

## 3. Results

We integrated a FE model mimicking RF ablation procedure with an optimization algorithm to predict input RF power and treatment time in order to achieve a value of VRR close to the VRR_target_, i.e., 65 percent [7]. Thus, we performed a parametric study for the active tip length of an 18-gauge RF electrode in the interval from 5 mm to 40 mm at the 5 mm step. In addition, we also analysed the influence of the blood perfusion rate of a thyroid nodule on the RF ablation treatment. It was evident from the available literature that the blood perfusion rate of thyroid nodules represents a difficult parameter to estimate, and thus, different values have been reported [31,40]. In this study, for each length of the RF electrode active tip, we performed simulations of the RF treatment for two values of blood perfusion, namely, ω_b_ = 0.0096 s^−1^ [31] and ω_b_ = 0.021 s^−1^ [40]. For all models, we applied the previously described optimization procedure.

**Figure 2:**
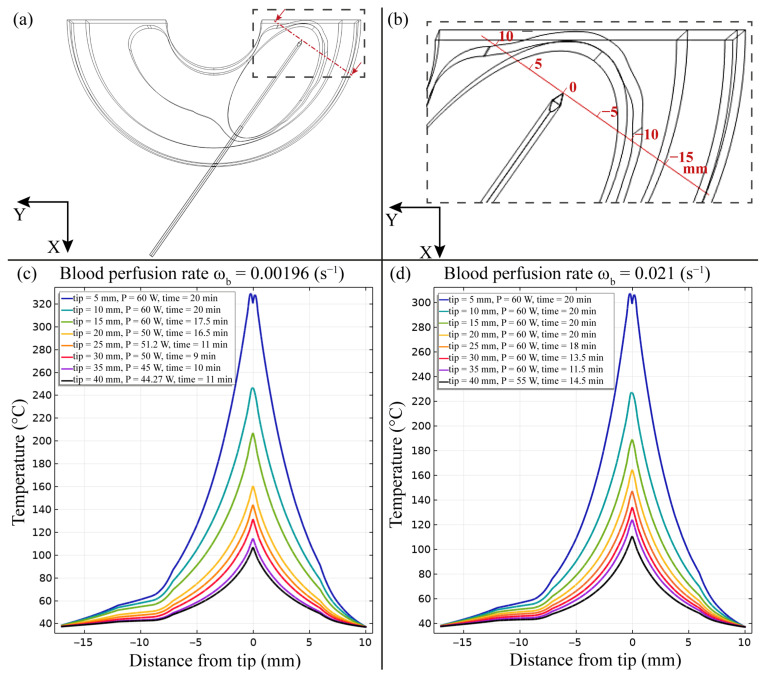
Temperature distribution obtained after RF ablation procedure as a function of distance from the electrode tip in the direction perpendicular to the electrode surface. (**a**,**b**) Schematic representation of the location measured (red line) for both conditions of blood perfusion rates, ω_b_ = 0.0096 s^−1^ (**c**) and ω_b_ = 0.021 s^−1^ (**d**).

The predicted effects of the RF ablation procedure were analysed in terms of temperature distribution near the RF electrode. Specifically, in Figure 2, we report the temperature distribution obtained as a function of the distance from the electrode tip in the direction perpendicular to electrode surface. Figure 2c shows the temperature trend for the condition with lower blood perfusion rate for different RF electrode active tip lengths. It is worth mentioning that, with an increase in active tips, the maximum temperature diminishes by up to three times with respect to the condition characterized by the minimum length of the active tip, i.e., 5 mm. Moreover, longer active tips lead to an up to 50 percent reduction in the transversal width of the ablation zone compared to the case with the 5 mm active tip. A similar trend is observed for the temperature profile and also for the condition with a higher blood perfusion rate (Figure 2d). In addition, in this case, it can be noticed that, for ω_b_ = 0.021 s^−1^, the maximum temperature achieved for all active tip lengths diminishes with respect to the corresponding outcomes of the condition characterized by ω_b_ = 0.0096 s^−1^. In fact, the increase in blood perfusion leads to a decrease in the local temperature. It is also observed for the ω_b_ = 0.021 s^−1^ condition that the transversal width of the ablation zone diminishes for longer active tips. However, in this case, a slightly modest reduction in the ablation width, i.e., up to 30 percent, is achieved in the condition with the 40 mm active tip length with respect to the model characterized by the 5 mm active tip length.

**Figure 3:**
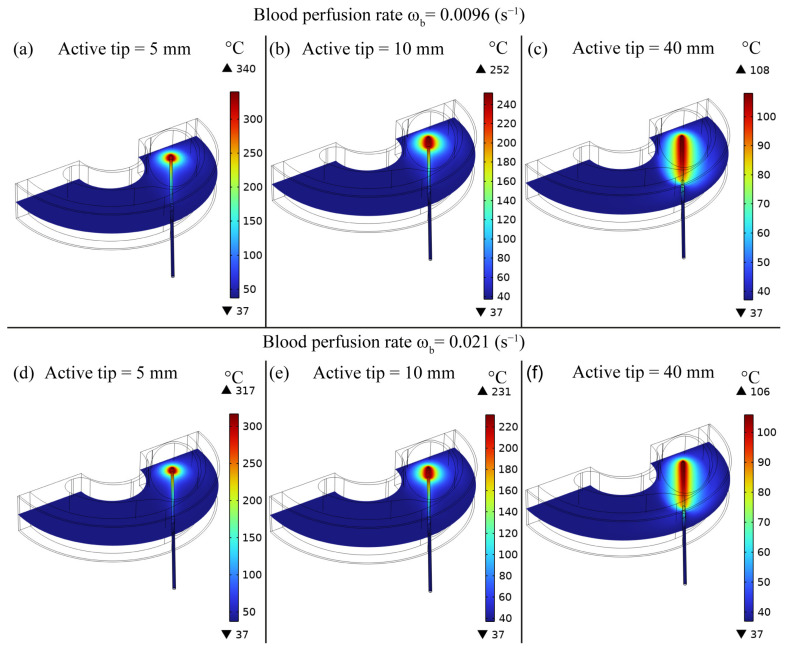
Temperature map obtained after RF ablation procedure in the central section (coordinate z = 10 mm) of the model characterized by active tip lengths of (**a**,**d**) 5 mm, (**b**,**e**) 10 mm, and (**c**,**f**) 40 mm for both conditions of blood perfusion rates, ω_b_ = 0.0096 s^−1^ (**a**–**c**) and ω_b_ = 0.021 s^−1^ (**d**–**f**).

In Figure 3, we illustrate the temperature map obtained after the RF ablation procedure in the central section (coordinate z = 10 mm) of the model characterized by the representative active tip lengths, i.e., 5 mm, 10 mm, and 40 mm, for both conditions of blood perfusion rates. All illustrated conditions show that the maximum temperature is achieved close to the active tip region, while it decreases at longer distances from the RF electrode. It can be noticed that, for longer active tips, the temperature range is reduced. Moreover, the temperature map highlights a diminution of the maximum temperature in the condition characterized by the higher blood perfusion rate.

**Figure 4:**
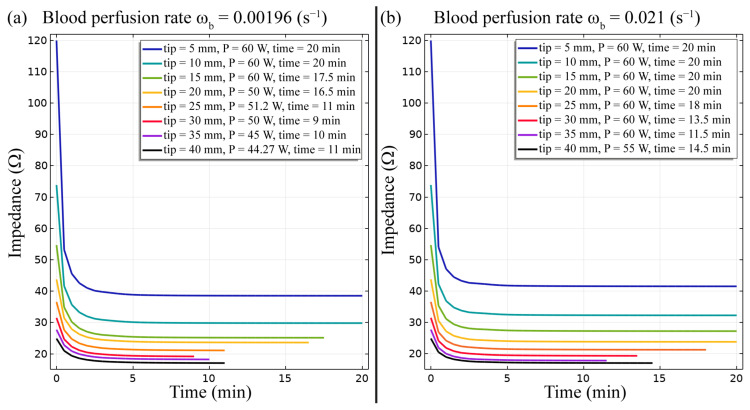
Impedance progress during the RF ablation procedure for different electrode active tip lengths for both conditions of blood perfusion rates, ω_b_ = 0.0096 s^−1^ (**a**) and ω_b_ = 0.021 s^−1^ (**b**).

The FE model allows to predict the evolution of the impedance during the RF ablation procedure (Figure 4). Overall, a decrease in the impedance can be observed during the initial period of the treatment. In addition, for both conditions of blood perfusion rates, a noticeable diminution of the impedance during the RF ablation treatment is achieved when smaller active tip length of the RF electrode is employed. For instance, a diminution of roughly 67 percent than the initial predicted value is observed in the case of ω_b_ = 0.0096 s^−1^ and of roughly 65 percent than the initial predicted value is observed in the case of ω_b_ = 0.021 s^−1^. Conversely, considering the longest active tip analysed in this study, i.e., 40 mm, the impedance is characterized by a modest decrease, e.g., roughly 30 percent lower than the initial predicted value in the case of ω_b_ = 0.0096 s^−1^ and roughly 28 percent lower than the initial predicted value in the case of ω_b_ = 0.021 s^−1^. The value of the blood perfusion rate also influences the impedance evolution. In fact, the condition with ω_b_ = 0.021 s^−1^ leads to a slightly minor decrease in the impedance with respect to the analogous case characterized by ω_b_ = 0.0096 s^−1^.

**Figure 5:**
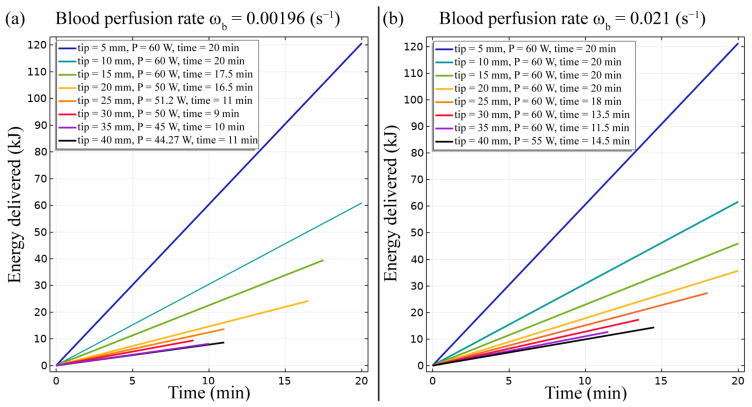
Energy delivered during the RF ablation procedure for different electrode active tip lengths for both conditions of blood perfusion rates, ω_b_ = 0.0096 s^−1^ (**a**) and ω_b_ = 0.021 s^−1^ (**b**).

In addition, as reported by [7], energy delivered is a significant parameter related to the efficacy of the treatment (Figure 5). As expected, the energy delivered increases with a higher duration of the treatment and a higher input power. The FE outcomes show that the energy delivered diminishes with the increasing length of the RF electrode active tip. In fact, the energy delivered in the case with an active tip of 40 mm length is lesser by roughly one order of magnitude than the energy estimated in the model with an active tip of 5 mm. 

**Figure 6:**
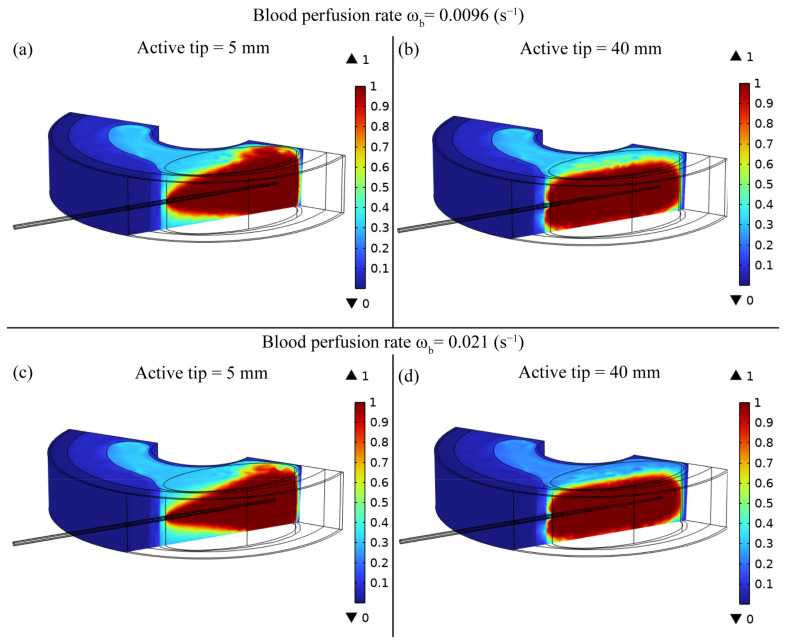
Tissue damage (θ) distribution obtained after RF ablation considering the model characterized by active tip lengths of (**a**,**c**) 5 mm and (**b**,**d**) 40 mm for both conditions of blood perfusion rates, ω_b_ = 0.0096 s^−1^ (**a**,**c**) and ω_b_ = 0.021 s^−1^ (**b**,**d**).

The fraction of thermal damage (θ) of the tissue due to the RF ablation is presented in Figure 6 for the two RF electrode active tip lengths of 5 mm and 40 mm and for the two blood perfusion conditions, i.e., ωb = 0.0096 s^−1^ and ωb = 0.021 s^−1^. In the case of an active tip of 5 mm, the RF therapy leads to complete thermal damage mainly in the region near the tip of the RF electrode. Moreover, in the case of the 5 mm active tip (Figure 6a,c), the damaged tissue also extends in the plane parallel to the electrode’s longitudinal axis. This outcome is also in agreement with the temperature distribution (Figure 2c) that shows a higher width of the ablation zone for smaller active tips. Conversely, in the case of a RF electrode active tip of 40 mm, the thermal damage occurs in a confined and uniform region along the electrode’s surface, without a remarkable extension in the plane parallel to the electrode’s longitudinal axis. 

**Figure 7:**
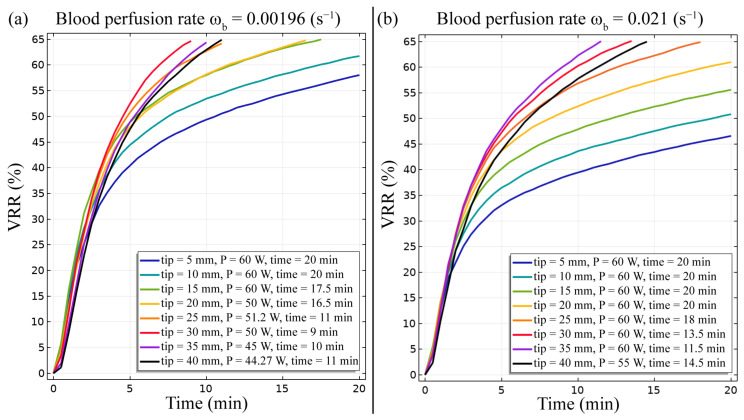
Volume reduction rate (VRR) achieved after the RF ablation procedure for different electrode active tip lengths for both conditions of blood perfusion rates, ω_b_ = 0.0096 s^−1^ (**a**) and ω_b_ = 0.021 s^−1^ (**b**).

Finally, the VRR predicted by means of the FE study is presented in Figure 7. We observe that values close to the VRR_target_ are achieved for the majority of the analysed conditions. In the case of blood perfusion rate equal to ω_b_ = 0.0096 s^−1^, the condition of electrode active tip between 5 mm and 15 mm requires a power of 60 W and a duration of the treatment of around 20 min. Conversely, for the models with RF electrode active tip length between 20 mm and 40 mm, the required power diminishes up to 44 W in the 40 mm active tip length case as well as the duration of the treatment is reduced to approximately 10 min. For the condition with a higher blood perfusion rate, the VRR succeeds in reaching the target value for lengths of the electrode active tip from 25 mm to 40 mm. The higher blood perfusion rate leads to a greater heat dispersion, and thus, the damaged tissue is reduced. Although maximum values of power and duration of treatment are adopted, the VRR values of almost 61 percent are achieved for active tip lengths lower than 20 mm. 

## 4. Discussion

RF ablation represents an efficient strategy in the treatment of thyroid nodules. In fact, several international guidelines [9,17,34] have suggested it as an appropriate alternative to surgical treatment in patients with benign thyroid nodules. The main concern in this therapy is to achieve an appropriate temperature in a tumour in order to achieve the optimal therapeutic effect with minimum damage to the healthy surrounding tissue. This aspect is still highly challenging to attain in practice, leading to the development of computational models [21,45,46] that allow to obtain insights into the RF ablation process. Although different aspects of RF ablation modelling are covered in the literature [21,23], the study of models for the identification of optimal technical RF parameters to achieve a specific target VRR still has not garnered enough attention. The present study combines recent advances on computational modelling to predict appropriate input power and treatment time that lead to a VRR close to the target value. A set of different conditions concerning the dimensions of the RF electrode active tip and the thyroid tumour blood perfusion rate have been analysed. 

The FE model outcomes allowed to predict and analyse the temperature distribution (Figure 2 and Figure 3), the impedance evolution (Figure 4), the energy delivered (Figure 5), the fraction of tissue damage (Figure 6), and the VRR (Figure 7) achieved after the RF ablation treatment. Overall, from the FE analysis, it is evident that longer active tips prevent damage occurrence in surrounding tissues since a reduced transversal ablation zone is developed with respect to the case characterized by smaller RF electrode active tips. In addition, models characterized by longer active tips predict reduced input power and treatment time to achieve target VRR, highlighting higher efficiency. In terms of treatment duration, the time is reduced by up to 50 percent for longer active tips. Overall, the minimum treatment time, i.e., 9 min, occurs in the case characterized by a blood perfusion rate of ω_b_ = 0.0096 s^−1^ and electrode active tip with length of 30 mm. The longest duration of the treatment and the highest power required to achieve a VRR value close to the target VRR is obtained in the case of blood perfusion rate ω_b_ = 0.021 s^−1^ and electrode active tip length of 25 mm.

From Figure 3, it can be observed that the maximum predicted temperature is achieved close to the electrode surface for all models. At tumour periphery, a significant variation in temperature is observed among models with different RF electrode active tip lengths. For instance, in the case of the 5 mm active tip length, the temperature at the periphery is roughly 37 °C. Conversely, the condition with a longer active tip, i.e., 40 mm, leads to a slightly higher temperature at the tumour periphery, e.g., an average of 45 °C. However, in this case, the temperature at the tumour periphery is below the required ablation temperature after the RF ablation treatment. 

Deandrea and colleagues [7] showed that the efficacy of the treatment was influenced by the delivered energy. A higher amount of delivered energy to the tumoral region leads to a higher performance of the RF ablation treatment. It is worth noticing that the amount of energy delivered in the case of 10 mm electrode active tip length is consistent with the previous clinical studies of Deandrea and co-workers [7,8].

The comparison among the outcomes predicted for the models characterized by different blood perfusion rates highlights that an increased power and treatment duration is required to achieve values closer to the target VRR for models characterized by ω_b_ = 0.021 s^−1^. As previously mentioned, for some conditions of small RF electrode active tip lengths considered in the highly perfused model, the maximum VRR achieved is smaller than the target VRR. In fact, the high perfusion rate inhibits energy deposition by removing the heat from the target volume and convecting it away to the surrounding tissue. Hence, to avoid longer treatment durations in a highly perfused hyperplastic thyroid nodule, the authors suggest that additional RF ablation sessions need to be taken into account in order to reach the target VRR.

This study presents several limitations that should be mentioned. Firstly, a simplified model of the neck anatomical region has been considered. Patient-specific images will be utilized in a future study to reconstruct the neck region in a more physiologically relevant manner. Secondly, the FE model assumed homogeneous tissue characterization. Values of tissues’ material properties are difficult to achieve. However, if accurate information concerning the spatial heterogeneity of physical parameters could be obtained, it could be subsequently used in the model to increase its accuracy [47]. Overall, the FE model is characterized by a trade-off among its complexity, which reflects the number of parameters to be calibrated, the computational processing power and the physiologically relevant representation of the anatomical area [24]. While many of these limitations will need to be overcome to enable clinical use, the model paves the way for patient-specific studies, allowing a better selection of parameters that minimize the damage to healthy surrounding tissues and decreases the time of the treatment. In addition, it is worth pointing out that the FE model does not take into consideration complex clinical scenarios, e.g., multiple nodules, since RF thermal treatment is not employed for similar cases [16,18].

## 5. Conclusions

RF ablation has emerged as a safe and effective treatment modality for benign thyroid nodules, offering a non-surgical alternative to traditional approaches. Experimental and computational models play a crucial role in achieving insights into the mechanisms underlying RF ablation of thyroid nodules. 

The present FE study simulates RF ablation operating at 480 kHz frequency. The electrode was found to play a significant role on the outcome of the RF ablation treatment. In particular, the analysis performed in this work evidenced that longer RF electrode active tips are more efficient to achieve higher VRR, minimizing the risk of damage for surrounding healthy tissues and diminishing the treatment duration. In fact, longer RF electrode active tip is characterized by a reduced transversal ablation zone. On the other hand, temperature achieves higher values with shorter RF electrodes.

Nonetheless, the study highlights that the electrode length is not the only factor that influences the process of RF ablation. In fact, the blood perfusion rate also has an effect on the parameters involved in the RF ablation process. Different combinations of RF electrode active tip lengths and blood perfusion rates lead to different outcomes in terms of RF powers and treatment times to reach VRR values close to VRR_target_. Comparison of the thermal response of the tissue suggested that RF electrode with longer active tips have the capacity to prevent overheating that allows a more controlled heating of tumour tissue.

The outcomes provided from the present 3D model may contribute to the optimization of the setting point of the RF ablation system in order to develop more appropriate patient-specific clinical protocols. Future research should integrate this algorithm with more physiologically relevant information, e.g., patient-specific geometry reconstructed from DICOM images, in order to develop a comprehensive understanding of benign thyroid nodule pathogenesis and facilitate the development of personalized diagnostic and therapeutic strategies.

## Data Availability

Data presented in this study are available on request from the corresponding authors.
